# Acute Activation of AMP-Activated Protein Kinase Prevents H_2_O_2_-Induced Premature Senescence in Primary Human Keratinocytes

**DOI:** 10.1371/journal.pone.0035092

**Published:** 2012-04-13

**Authors:** Yasuo Ido, Albert Duranton, Fan Lan, Jose M. Cacicedo, Tai C. Chen, Lionel Breton, Neil B. Ruderman

**Affiliations:** 1 Section of Endocrinology and Diabetes Research Unit, Department of Medicine, Boston University School of Medicine, Boston, Massachusetts, United States of America; 2 L'OREAL Recherche, Centre Charles Zviak, Clichy, France; University of Medicine and Dentistry of New Jersey, United States of America

## Abstract

We investigated the effects of AMPK on H_2_O_2_-induced premature senescence in primary human keratinocytes. Incubation with 50 µM H_2_O_2_ for 2 h resulted in premature senescence with characteristic increases in senescence-associated ß-galactosidase (SA-gal) staining 3 days later and no changes in AMPK or p38 MAPK activity. The increase in SA-gal staining was preceded by increases in both p53 phosphorylation (S15) (1 h) and transactivation (6 h) and the abundance of the cyclin inhibitor p21^CIP1^ (16 h). Incubation with AICAR or resveratrol, both of which activated AMPK, prevented the H_2_O_2_-induced increases in both SA-Gal staining and p21 abundance. In addition, AICAR diminished the increase in p53 transactivation. The decreases in SA-Gal expression induced by resveratrol and AICAR were prevented by the pharmacological AMPK inhibitor Compound C, expression of a DN-AMPK or AMPK knock-down with shRNA. Likewise, both knockdown of AMPK and expression of DN-AMPK were sufficient to induce senescence, even in the absence of exogenous H_2_O_2_. As reported by others, we found that AMPK activation by itself increased p53 phosphorylation at S15 in embryonic fibroblasts (MEF), whereas under the same conditions it decreased p53 phosphorylation in the keratinocytes, human aortic endothelial cells, and human HT1080 fibrosarcoma cells. In conclusion, the results indicate that H_2_O_2_ at low concentrations causes premature senescence in human keratinocytes by activating p53-p21^CIP1^ signaling and that these effects can be prevented by acute AMPK activation and enhanced by AMPK downregulation. They also suggest that this action of AMPK may be cell or context-specific.

## Introduction

Ageing is a physiological phenomenon that occurs in all eukaryocytes. In many tissues, it is associated with an increased number of senesced cells [1], [2]. Although senescent cells undergo an apparently irreversible growth arrest, they remain metabolically active and display characteristic changes in cell morphology, physiology and gene expression and a resistance to apoptosis. In addition, they produce inflammatory and other factors that can have adverse effects on adjacent cells that in turn could contribute to various disorders [1]. Recently it has been reported that elimination of senescent cells in a mouse with a progeroid background delays the development of an aging phenotype in various organs [3]. Thus, cellular senescence could also play a critical role in aging in vivo.

Replicative senescence, first described in cultured human fibroblasts 50 years ago [4], is the result of erosion of telomeres and occurs at every cell division. The definition of senescence has since been broadened, and it is now accepted that a senescence phenotype can occur irrespective of telomere status [5]. This so called “premature” senescence may be caused by oxidative and other stresses that cause DNA damage, chromatin perturbation (e.g. by histone acetylation), oncogenes and other factors [1]. It has also been established that the activation of p53 and p38 MAPK, and subsequent to this the induction of their cyclin-dependent kinase (CDK) inhibitors, p21^CIP1^ and p16^INK4a^, can be key factors in producing and maintaining growth arrest in these cells [6]. A defining characteristic of cells with both replicative and premature senescence is an increase in the staining of senescence-associated β-galactosidase (SA-Gal) [2].

Skin is the largest organ in the body and its epidermis is self-renewing and metabolically very active. Epidermis, especially of skin on the face, is exposed to UV light that leads to oxidative stress and DNA damage that, in turn, predispose it to various diseases including cancers, as well as to changes that give it the characteristic appearance of aging [7]. In keeping with this, senescent cells, positive for SA-Gal activity, were first described in vivo in the epidermis of aged humans [2]. Also, exposure of keratinocytes in vitro to low doses of hydrogen peroxide, twice in a 5 day period, has been shown to induce premature senescence, as evidenced by decreased population doublings and increased SA-Gal positive cells [8]. In the studies described here, we used a similar model in which keratinocytes were exposed only once to H_2_O_2_ to examine the effects of the fuel sensing and signaling molecule AMP-activated protein kinase (AMPK) on these events.

AMPK is a heterotrimeric enzyme that consists of α, β and γ subunits [9]. It initially drew attention because of its role in sensing a cell's energy state and restoring it to normal values when low. Thus, increases in the AMP/ATP ratio (low energy state) have repeatedly been shown to lead to AMPK activation, which in turn activates various processes that increase ATP generation (e.g. fatty acid oxidation, mitochondrial function) and decreases others that consume ATP, but can be downregulated without compromising the cell (e.g. protein and lipid synthesis, cell growth and proliferation) [10]. More recent studies suggest that AMPK plays a much wider role in the regulation of cellular function. In particular it appears to be a major factor in combating many stresses. For instance, in human endothelial cells, activation of AMPK by AICAR (aminoimidazole carboxamide ribonucleotide) and other agents has been shown to prevent the apoptosis, mitochondrial dysfunction, insulin resistance, inflammation and oxidative stress caused by hyperglycemia [11], [12], [13], the fatty acid palmitate [14]; and TNFα [14], [15]. In addition, in various tissues AMPK has been shown to affect genes that regulate mitochondrial function [16]; the expression of anti-oxidant enzymes [17]; the phosphorylation of transcriptional factors such as p53 and the FOXOs [18], [19]; cellular autophagy [20], [21] and polarity [22], [23] and the activity of SIRT1, a histone protein deacetylase that has been linked to increased longevity [24]. Furthermore AMPK appears to regulate, at least in part, the therapeutic benefits of exercise and a variety of pharmacological agents (e.g. metformin, thiazolidinediones) in experimental animals and where studied in humans [25], [26].

Despite its many actions, the effects of AMPK on cellular senescence have received relatively little attention. In two widely quoted studies, this question was examined in cultured fibroblasts. In one of them, Jones et al [19] found that AICAR treatment or overexpression of a CA-AMPK in mus musculus embryonic fibroblasts (MEF) led to p53 phosphorylation on S15 and growth arrest within hrs and over a longer period of time (days) to increased SA-Gal staining. They also found that growth arrest and enhanced SA-Gal activity did not occur in MEFs lacking p53. A similar effect of prolonged AMPK activation (3–7 days) on senescence was also reported in human fibroblasts incubated with pharmacological AMPK activators (AICAR, antimycin A, azide) by Wang et al [27]. In this study, replicative senescence was associated with an increase in the AMP/ATP ratio, and increased p16, as well as activation of AMPK. The phosphorylation of p53 was not studied.

In light of the above findings, we have examined the effects of AMPK on cellular senescence in keratinocytes. For this purpose, primary human keratinocytes were incubated with a low dose of H_2_O_2_ that induces a senescent phenotype [8]. We examined the events associated with the development of senescence in these cells, and in particular whether AMPK activity is altered and/or its activation attenuates or accelerates the senescence. In addition, we compared the effects of AMPK on p53 phosphorylation, a key event in the development of senescence, under identical conditions in keratinocytes, MEFs and other cells.

## Results

### Establishment of conditions for inducing premature senescence in primary human keratinocytes

**Figure 1:**
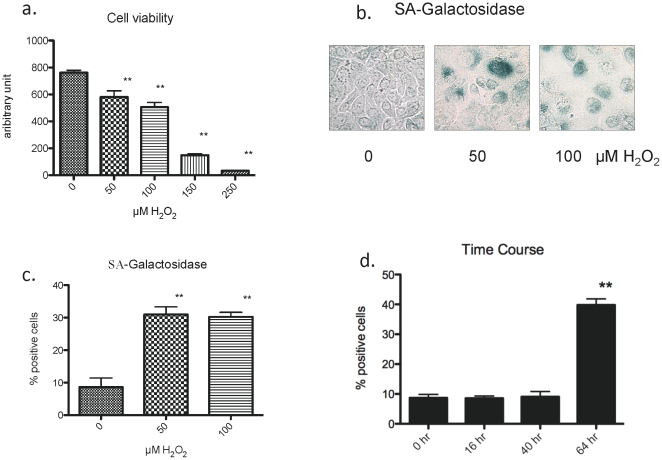
Effects of H_2_O_2_ on cell viability and senescence. **a.** Cell viability was assessed by alamar blue fluorescence (Ex 530, Em. 590 nm) 16 hr after the addition of H_2_O_2_ at the indicated concentrations (n = 6, ** p<0.01). Incubation with H_2_O_2_ at concentrations between 50–250 µM progressively decreased cell viability. **b.** SA-galactosidase (SA-Gal) activity. Cells were incubated in media containing the indicated concentration of H_2_O_2_ for 2 hr. They were then incubated in the same medium without H_2_O_2_ for 3–4 days, after which they were stained for SA-Gal. H_2_O_2_ increased the number of positive cells. **c.** Percentage of positive cells was increased approximately 3-fold by prior incubation with 50 or 100 µM H_2_O_2_. In excess of 300 cells were counted for each condition. (n = 3–6, ** p<0.01) **d.** Time course of appearance of SA-galactosidase positive cells after treatment with 50 µM H_2_O_2_.

Previous reports suggest that keratinocytes may undergo apoptosis or premature senescence depending on the dose of H_2_O_2_ to which they are exposed [8], [28]. Thus, we initially examined the effects of different doses of H_2_O_2_ on cell viability. The alamar blue assay was used to measure viability 16 hours after a 2 hr exposure to H_2_O_2_ at the indicated concentrations (see Materials and Methods). As shown in Figure 1a, increasing the concentration of hydrogen peroxide progressively decreased cell viability, and after exposure to 250 µM for 2 hrs virtually no viable cells were observed at 16 h. Next we assessed whether exposure to lower doses of hydrogen peroxide induces premature senescence as previously reported [8] and if so, at what time post exposure. H_2_O_2_ at a dose of 50 or 100 µM was added to the culture medium for 2 h at day 3 post seeding and the cells were refed standard growth medium and cultured up to 8 days. By 3–4 days post H_2_O_2_ exposure, the keratinocytes treated with these concentrations of H_2_O_2_ were viable, variable in size and less densely populated than control cells. In addition, strong positive staining for SA-Gal was seen in some, but, not all cells (Fig. 1b), with the percentage of SA-Gal positive cells increased by 3.5 fold (p<0.01) (Fig. 1c). These experiments were repeated several times with keratinocytes from different individuals and passaged up to 4 times with similar results. As shown in Fig. 1d, incubation with 50 µM H_2_O_2_ for 2 hrs did not increase SA-Gal staining after 16 or 40 hrs, but did so at 64 hrs. Likewise, we obtained identical findings when H_2_O_2_ was added twice, once at 3 days post seeding for 2 hours and again once at 5 days post-seeding for 2 hours, in keeping with the findings of Bernard et al. [8]. Since a single exposure of H_2_O_2_ was sufficient to cause senescence 64 hrs post exposure, we used this model in subsequent studies.

### Assessment of the expression of AMPK upstream regulators and downstream targets when hydrogen peroxide induces senescence

**Figure 2:**
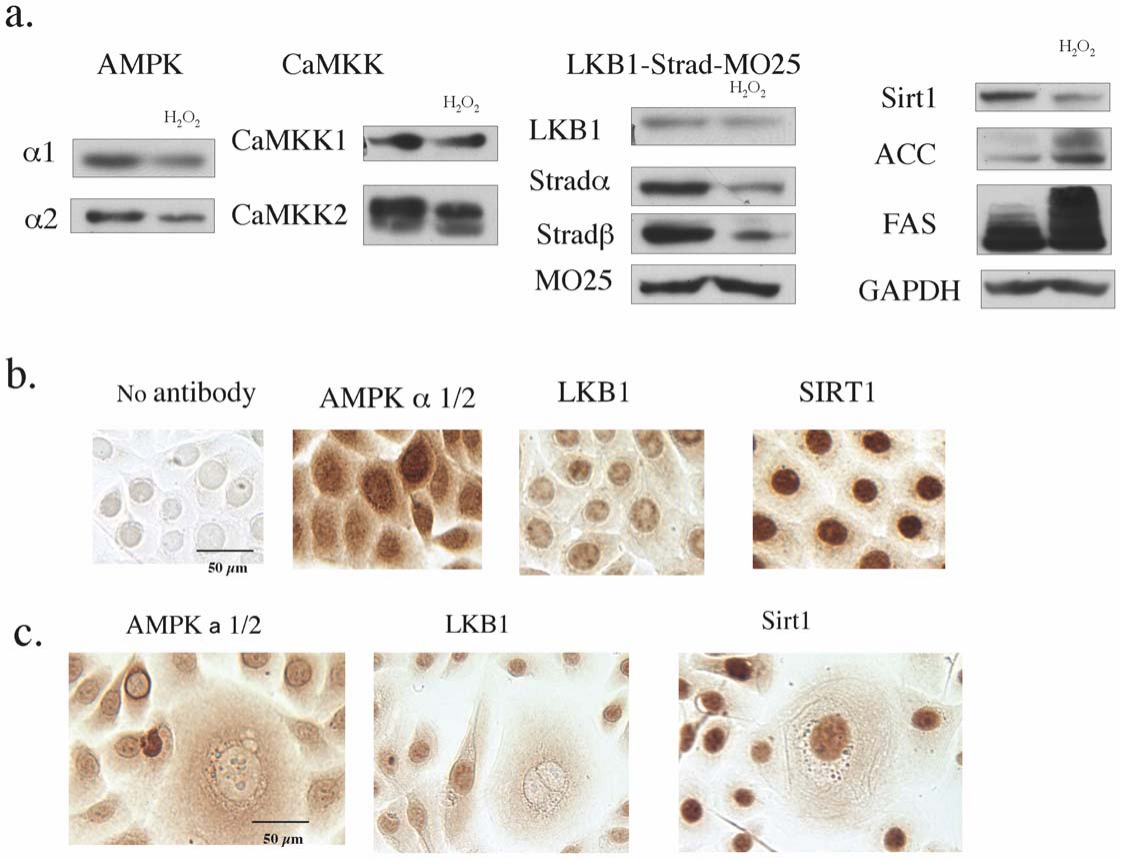
Effects of H_2_O_2_ on the expression of AMPK and some of its upstream regulators and down-stream targets. **a. Western blot analysis.** Cells were grown on a 6 well plate. In three wells they were treated with 50 µM H_2_O_2_ for 2 hrs, 5 days after seeding to produce senescence. Sixty four hours after exposure to H_2_O_2_, the treated and control cells were harvested for SDS-PAGE and western blotting. After adjusting for differences in protein concentration, lysates from 3 wells in the same treatment group were combined to minimize variation and proteins were separated on 4–12% gradient gels and then transferred to a PDVF membrane. In keratinocytes treated with H_2_O_2_, the expression of AMPK alpha 2, CaMKK2, Strad alpha and beta and SIRT1 were all diminished. Modest decreases in AMPK alpha 1, and LKB1 expression were also observed, whereas CaMKK1 and MO25 were not affected. Thus the AMPK system was down-regulated in senesced cells. In agreement with this conclusion, both ACC and FAS expression were increased. Results are representative of 3 measurements. **b, c. Immunocytochemical analysis of AMPK, LKB1 and SIRT in control (b) and H_2_O_2_ treated cells at 64 hr (c).** Control cells and cells treated with 50 µM hydrogen peroxide were incubated as described in 2a. At sixty four hours, they were fixed with 2% paraformaldehyde and incubated with the indicated primary antibodies and detected with the UltraTech HRP kit and NovaRed. Figures in (c) were selected to show enlarged-senescent cells and control cells. AMPK staining was observed in the nucleus as well as the cytosol, suggesting the presence of both its alpha 1 (cytosol) and alpha2 (cytosol and nucleus) isoforms. Both LKB1 and SIRT1 were predominantly stained in the nucleus. (scale bar = 50 µm). Following H_2_O_2_ treatment the nuclear localization of AMPK and LKB1, but not SIRT1, was lost in senesced cells (c). The photomicrographs in (c) were selected to show enlarged senescent cells and are not representative of the population as a whole, as thet are in Figures 1,3 (scale bar = 100 µm). Results are representative of 3 experiments.

Primary keratinocytes possess a functional AMPK cascade that is upregulated by AICAR, rosiglitazone and increases in cell density [29]. In the present study, we found that primary human keratinocytes express both the alpha 1 and 2 isoforms of AMPK, with alpha1 the dominant isoform (see below), as well as the two major AMPK kinases, CaMKK and LKB1-STRAD-MO25 (Fig. 2a). The results also revealed that LKB1 and SIRT1, which can be an upstream regulator of LKB1 ([30] and see below), are predominantly localized in the nucleus (Fig. 2b).

Sixty-four hrs after a single 2 hour treatment with 50 µM hydrogen peroxide, i.e., when the senescence phenotype was observed, the expression of AMPK alpha 2, CaMKK2, Strad alpha and beta and SIRT1 were all markedly decreased (Fig. 1a). AMPK alpha 1 and LKB1 were moderately decreased and CaMKK1 and MO25 unchanged. Thus, overall the AMPK signaling system was down-regulated in senesced cells. In keeping with this conclusion, the protein expression of various AMPK targets, including both ACC (acetyl-CoA carboxylase) and FAS (fatty acid synthase), which AMPK transcriptionally downregulates, was increased and that of the house-keeping gene GAPDH was unchanged (Fig. 2a). Immunocytochemistry revealed a loss of nuclear LKB1 and AMPK; however, SIRT1 staining was not noticeably altered (Fig. 2c).

### Down-regulation of AMPK by expression of dominant negative (DN)-AMPK causes a senescent-like phenotype

**Figure 3:**
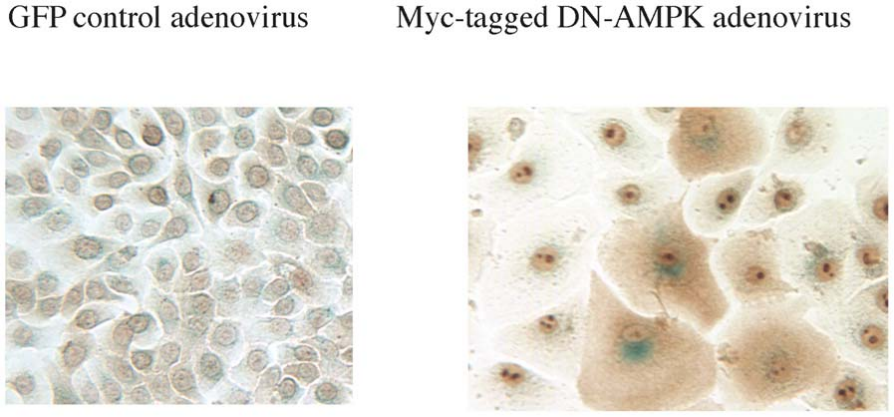
Adenovirus mediated gene transfer and the effects of dominant negative (DN) AMPK expression on SA-Gal staining and morphology. Cells were infected with recombinant adenovirus expressing myc-tagged DN-AMPK 4 days after seeding. Immunohistochemical staining with myc-antibody was performed following SA-Gal staining. Infected cells were easily detected by cytosolic staining with myc-antibody. SA-Gal staining was diminished by repeated washing during immuno-staining but was still detectable. Almost all infected cells were enlarged and stained positively for SA-Gal.

We next attempted to evaluate the effects of AMPK down-regulation on primary human keratinocytes in the absence of H_2_O_2_. As observed with many other primary cells, the keratinocytes were not easily transfected by traditional lipid-based agents. Thus, we chose to use recombinant viruses. In preliminary experiments, three different recombinant virus systems were evaluated; adenovirus [31], adeno-associated virus (AAV type 2), and lentivirus. Viruses expressing GFP proteins were tested to assess the feasibility of using each of them. We found that human keratinocytes can be infected with all three. Infection of GFP expressing adenovirus and AAV had no effect on cell growth or morphology or SA-Gal staining compared to non-infected cells and GFP expression was evident for at least 1 week (Fig. 3 and Fig. S1).

Based on these results, we evaluated the effects of infection with recombinant adenovirus expressing myc-tagged dominant negative (DN) alpha1-AMPK. Such keratinocytes were easily detected by immunocytochemical staining of their cytosol with myc-antibody (Fig. 3). SA-Gal staining was still visible, although its intensity was diminished because of repeated washing during immunostaining. Almost all DN-AMPK positive cells were SA-Gal positive and larger in size, suggesting that endogenous AMPK activity regulates senescence in the keratinocyte. Similar findings, obtained when keratinocytes were infected with a lentivirus expressing shRNA targeting AMPKα1, will be presented later in conjunction with studies with AMPK activators.

### Activation of AMPK by AICAR or resveratrol prevents hydrogen-peroxide induced senescence

**Figure 4:**
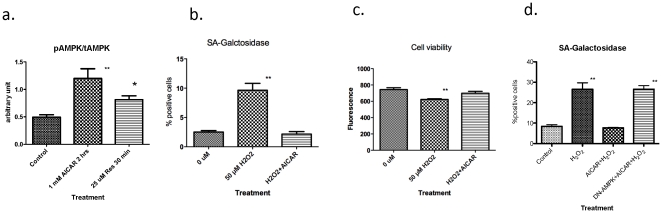
Effects of AMPK activation by AICAR (1 mM) on H_2_O_2_ induced SA-galactosidase expression. **a.** AICAR increased Thr172 phosphorylation of AMPK in 2 h and resveratrol in 30 min–2 hrs. (n = 3, * p<0.05, ** p<0.01) **b.** Effects of AICAR on SA-Gal activity. Cells were treated with 1 mM AICAR 2 hrs prior to exposure to 50 µM H_2_O_2_. SA-Gal activity was assessed 64 hrs later. **c.** Cells were treated as in **b.**, except that after 16 hrs cell viability was assessed after incubation with alamar blue for 1 hr (n = 3–6, ** p<0.01). **d.** Effect of dominant negative (DN)-AMPK adenovirus infection on the suppression of H_2_O_2_ induced SA-galactosidase expression by AICAR. DN-AMPK expressing adenovirus was added to the cells 24 hrs before the experiment. (n = 3, ** p<0.01 vs control).

We next assessed the effect of AMPK activation on hydrogen peroxide induced premature senescence. AICAR, a widely used AMPK activator, and resveratrol, a putative direct activator of both AMPK and SIRT1 [32] were used. AICAR activates AMPK when it is converted in cells to ZMP (5-aminoimidazole-4-carboxamide-1-β-D-ribofuranosyl-5-monophosphate), an AMP analog, and resveratrol is thought to do so both by activating SIRT1, which deacetylates LKB1, leading to its activation and then to that of AMPK [30] and by a direct action on mitochondria that leads to a charge in cellular energy state. As shown in (Fig. 4a), both AICAR and resveratrol activated AMPK in 2 hours or less.

We added AICAR 1 hour before hydrogen peroxide treatment and, as in other studies, replaced the basic medium with growth medium after 2 hours of H_2_O_2_ exposure. As shown in Fig. 4b, after 64 hr, prior incubation with 50 µM H_2_O_2_ caused a 4-fold increase in the percentage of SA-Gal positive cells (2.5±0.26% (control) vs 9.6±1.2%, n = 6, p<0.001), and AICAR totally prevented this (2.2±0.4%). Cell viability assessed with alamar blue was slightly decreased in cells treated with 50 µM hydrogen peroxide (p<0.05, n = 3) and this too was prevented by AICAR (Fig. 4c). In a separate experiment, we tested whether prevention of AMPK activation by infection with a dominant negative (DN)-AMPK, 2 days prior to the experiment, affected AICAR's ability to prevent senescence. As shown in Fig. 4d, it totally eliminated AICAR's ability to do so, further indicating that its protective action was AMPK mediated.

**Figure 5:**
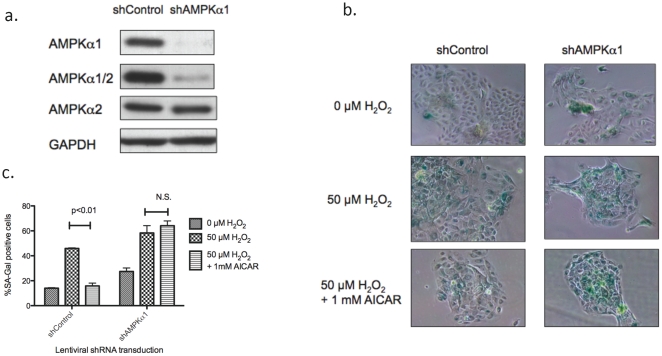
Effects of AMPK knock-down with shAMPKα1 on SA-galactosidase expression. **a.** Lentivirus vectors expressing shRNA targeting human AMPK alpha subunit were created. One of them knocked-down the AMPK alpha1 subunit more than 95%, 48 hours after infection, without affecting alpha2 expression. Total AMPK alpha was suppressed more than 80%. **b, c.** Keratinocytes grown in 12 well plates were infected with lentivirus expressing control (non-targeting) shRNA (shControl) or AMPK alpha1 (shAMPKα1) 48 hours prior to incubating for 2 hours with H_2_O_2_±1 mM AICAR SA-Gal activity was assessed 64 hours later. In fection of the cells with shAMPKα1 both significantly increased basal SA-Gal (*, p<0.05 vs shControl+0 mM H_2_O_2_, n = 4) and eliminated the preventive effects of AICAR (†, p<0.05 vs shAMPKα1+0 mM H_2_O_2_, n = 4).

To evaluate further the effects of AMPK on premature senescence we performed AMPK knock-down experiments using short interference (si) or short hairpin (sh) RNA. Transfection of commercially prepared siRNA resulted in unsatisfactory decreases in both AMPK alpha 1 and 2 (not shown), likely due to difficulty in its transfection in primary keratinocytes. Thus, we made lentivirus vectors expressing shRNA to either alpha1 or alpha2. Among 3 vectors created (2 for alpha1 and 1 for alpha2), we found one which targeted alpha1 that suppressed AMPK alpha1 by more than 95% and total AMPK by 80–90% (Fig. 5a.) 48 hrs after transduction. Subsequently, we infected keratinocytes with lentiviruses expressing shRNA targeting a non-human sequence (shControl) or AMPK alpha1 (shAMPKα1) 48 hours before applying H_2_O_2_. As shown in Fig. 5 bc, knock-down of AMPK by itself resulted in a two-fold increase in SA-Gal positive cells and it eliminated the effects of AICAR completely.

**Figure 6:**
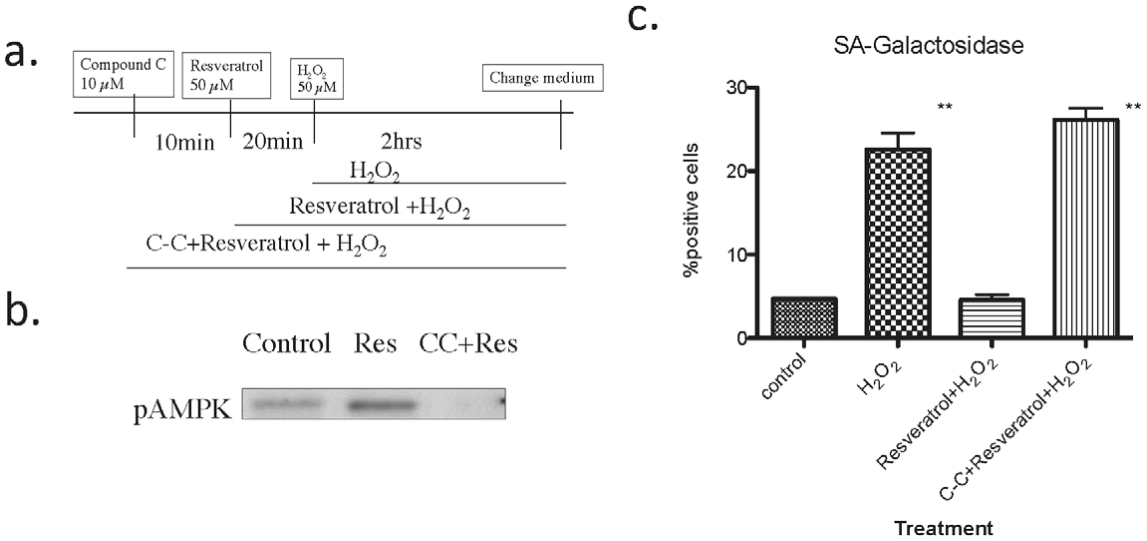
Effects of resveratrol and the AMPK inhibitor Compound C on H_2_O_2_ induced SA-galactosidase expression. **a.** Basic experimental design. **b.** AMPK activation by resveratrol and its inhibition by Compound C. **c.** Inhibition of H_2_O_2_ induced senescence by resveratrol and its prevention by Compound C. SA-Gal staining was performed 64 hrs after the cessation of H_2_O_2_ treatment (n = 3, ** p<0.01 vs control).

The effects of resveratrol, added 30 min prior to H_2_O_2_, were studied in the presence and absence of the AMPK inhibitor Compound C [33](Fig. 6a). As shown in the western blot, Compound C completely eliminated the increase in phospho T172-AMPK caused by resveratrol (Fig. 6b) as well as its ability to prevent H_2_O_2_ induced cellular senescence (Fig. 6c). Thus, although resveratrol may have other effects, AMPK activation is crucial for its ability to inhibit H_2_O_2_ induced senescence.

### P53, but not p38MAPK or AMPK, is activated by low doses of hydrogen peroxide

**Figure 7:**
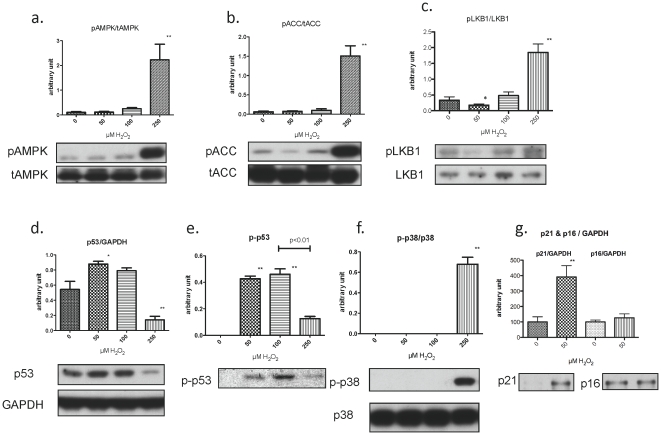
AMPK, LKB1, p53, p21, and p16 protein expression after H_2_O_2_ exposure for 1 and 16 hr. Keratinocytes were treated with the indicated concentration of H_2_O_2_ and harvested after 1 hr (**a–f**) or16 hr (**g**.) for western blotting (**a,b**). AMPK activity, assessed by its phosphorylation at Thr172 and that of its substrate ACC Ser79, was increased only at the high (250 µM) concentration of H_2_O_2_(**c**). Phosphorylation of LKB1 (Ser431), an upstream kinase of AMPK, was slightly decreased by 50 µM H_2_O_2_ and increased by 250 µM H_2_O_2_ (**d, e**). p53 expression and phosphorylation were increased by 50 and 100 µM H_2_O_2_ and were variably affected by 250 µM H_2_O_2_ (**f**). Activation (phosphorylation) of p38-MAPK was only observed with 250 µM H_2_O_2_. **g.** Incubation with H_2_O_2_ (50 µM) for 16 h induced the cyclin-dependent kinase inhibitor, p21^CIP1^ but not p16 ^INK^. (n = 3–6, * p<0.05, ** p<0.01).

We examined the effects of H_2_O_2_ at various concentrations on the phosphorylation and/or abundance of AMPK, p53, p21 and LKB1 in primary keratinocytes. When studied 1 hr after the addition of 50 or 100 µM H_2_O_2_, AMPK activity, assessed both by the phosphorylation of its alpha subunit at T172 (Fig. 7a) and of its substrate ACC (acetyl-CoA carboxylase, Fig. 7b), was not substantially altered, nor was the phosphorylation of the AMPK kinase LKB1 at S431. In contrast, the phosphorylation of all of these molecules was increased after incubation with 250 µM H_2_O_2_ (Fig. 7c).

In contrast to the lack of change in AMPK, ACC and LKB1, p53 abundance at 1 h was increased by 50% at 50 and 100 µM H_2_O_2_, but was decreased at 250 µM (Fig. 7d). Also p53 phosphorylation at S15, an index of its activation, was dramatically increased by 50 and 100 µM H_2_O_2_ and to a lesser extent by 250 µM H_2_O_2_ (Fig. 7e). No detectable p38MAPK phosphorylation was observed at 50 and 100 µM H_2_O_2_, even after 24 hrs (not shown); but, such phosphorylation was strongly increased at 250 µM H_2_O_2_ (Fig. 7f). Finally, incubation with a low dose of H_2_O_2_ (50 µM) increased the abundance of p21^CIP1^, but not p16 at 16 h (Fig. 7g). Taken together, these findings indicate that low-doses of H_2_O_2_ activate p53, but not AMPK or p38MAPK. They also suggest that the senescence induced by low dose hydrogen peroxide is caused by activation of the p53-p21^CIP1^, but not the p38MAPK-p16^INK4a^ cascade.

### H_2_O_2_-induced increase in p21^CIP1^ requires p53 and is up-regulated by AMPK knock-down

**Figure 8:**
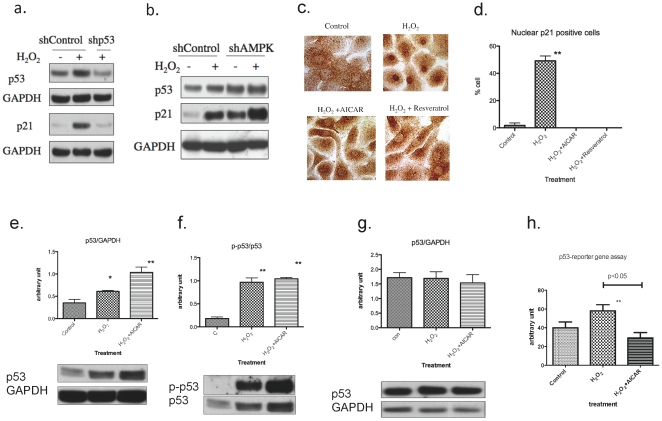
Effects of p53 or AMPK knock-down and AMPK activation on p21 and p53 expression. **a. Effects of p53-knockown on p53 and p21^CIP1^expressions.** Cells grown on 6 well plates were infected with lentiviral vector, expressing short-hairpin (sh) RNA targeting non-human sequence (Control) or p53, at 30% confluence. Forty-eight hours later, cells were treated with 50 µM H_2_O_2_ for 2 hr, and harvested after 1 hr (for p53 expression) or 16 hours (p21^CIP1^ expression). The knock-down of p53 completely blocked H_2_O_2_ induced upregulation of p53 and subsequent in p21^CIP1^. Experiments were performed in duplicate and representative blots are shown. **b. Effects of AMPK knock-down on p53 and p21^CIP1^ expression.** AMPK knock-down effects were assessed as in the preceding experiment. Cells were harvested at 16 hr after H_2_O_2_ treatment. AMPK knock-down increased both p53 and p21^CIP1^ expression and H_2_O_2_ increased p21^CIP1^ further. Experiments were performed in duplicate and a representative blots is shown. The result is consistent with the increased SA-Gal expression casued by AMPK knock-down as shown in fig. 5. **c, d. Effects of AICAR and resveratrol on H_2_O_2_ induced p21^CIP1^ expression.** (**c.**) Cells grown on 6 well plates were treated with 50 µM H_2_O_2_ for 2 hr with/without 30 min pretreatment with 50 µM resveratrol or 1 mM AICAR. After sixteen hours of incubation, in standard growth medium, the cells were fixed with 2% paraformaldehyde and immunostained for p21^CIP1^. H_2_O_2_ significantly increased positive staining in the nucleus (non-specific staining can be seen in cytosol), and this was largely prevented by AICAR and resveratrol. Scale bar = 50 µm. (**d.**) Quantitation of 150–200 cells from (a) revealed that approximately 50% of cells treated with hydrogen peroxide exhibited predominantly nuclear staining of p21^CIP1^ and that AICAR and resveratrol completely prevented this from occurring. (n = 3, ** p<0.01). **e–f. Effects of AICAR on H_2_O_2_-induced p53 expression and phosphorylation.** (**e.**) p53 phosphorylation was increased by H_2_O_2_ exposure and this was not affected by AICAR (n = 3, ** p<0.01 vs 6). (**f.**) Cells were incubated with 1 mM AICAR for 1 hr prior to addition of 50 µM H2O2 and then harvested 1 hr later. p53 protein abundance was increased by H_2_O_2_ and AICAR had an additive effect on this. **e.** Increases in p53 abundance in H_2_O_2_ only and H_2_O_2_+AICAR groups observed at 1 hr were not observed at 16 hr. **g. p53 reporter gene assay.** The cells were infected with a p53-luciferase reporter adenovirus. Twenty-four hours later, one group was pre-treated for 1 hr with 1 mM AICAR then given 50 µH_2_O_2_. Six hours later the cells were harvested and luciferase activity assessed. Luciferase activity, which reflects p53 transactivation, was significantly increased by H_2_O_2_ and was suppressed by 1 mM AICAR. (n = 4, p<0.05 for both).

Since p21^CIPI^ is a well-known target of p53, induction of p21^CIPI^ by H_2_O_2_ is likely the result of p53 activation. In keeping with this conclusion, when p53 was knocked-down by infecting keratinocytes with a shRNA to p53 (shp53) expressing lentiviral vector, the increase in p53 protein caused by H_2_O_2_ was prevented and the induction of p21^CIP1^ was completely inhibited (Fig. 8a), We also examined effects of AMPK knock-down on p53 and p21^CIPI^. Consistent with the results shown in fig. 5, AMPK knock-down strongly induced p21^CIPI^ with a modest increase in p53 (Fig. 8b).

### Inhibition of p21^CIP1^ expression by AMPK is associated with decreased p53 transactivation

The increase in the abundance and nuclear localization of the p21^CIPI^ preceded the increase in SA-Gal in keratinocytes incubated with 50 µM H_2_O_2_ (16 h vs 40–64 h). The data in Fig. 8c, d, show that a brief period of exposure to AICAR or reservatrol (30 mm) totally prevents this increase in nuclear p21^CIPI^. To evaluate whether this effect of AICAR was mediated by p53 we determined p53 phosphorylation at 1 hr and its abundance at 1 hr and 16 hr post H_2_O_2_ treatment. In addition, its p53 transactivation (assessed by the fire-fly luciferase reporter gene assay) was determined 6 hr after the addition of hydrogen peroxide. As shown in Fig. 8e, f, at 1 h AICAR did not diminish the increase in p53 phosphorylation (S15) and it modestly enhanced the increase in p53 abundance caused by H_2_O_2_. 16 h post- H_2_O_2_, p53 abundance was neither increased compared to a control group (no H_2_O_2_) nor was it altered by AICAR (Fig. 8g). Finally, p53 transactivation was increased 16 h after H_2_O_2_ treatment and preincubation with AICAR totally prevented this (Fig. 8h). Thus, it appears that AMPK could act initially by inhibiting p53 transactivation and subsequent to this p21^CIP1^ induction when it prevents the premature senescence caused by low doses of H_2_O_2_.

### Activation of AMPK by AICAR does not induce phosphorylation of p53 in keratinocytes

**Figure 9:**
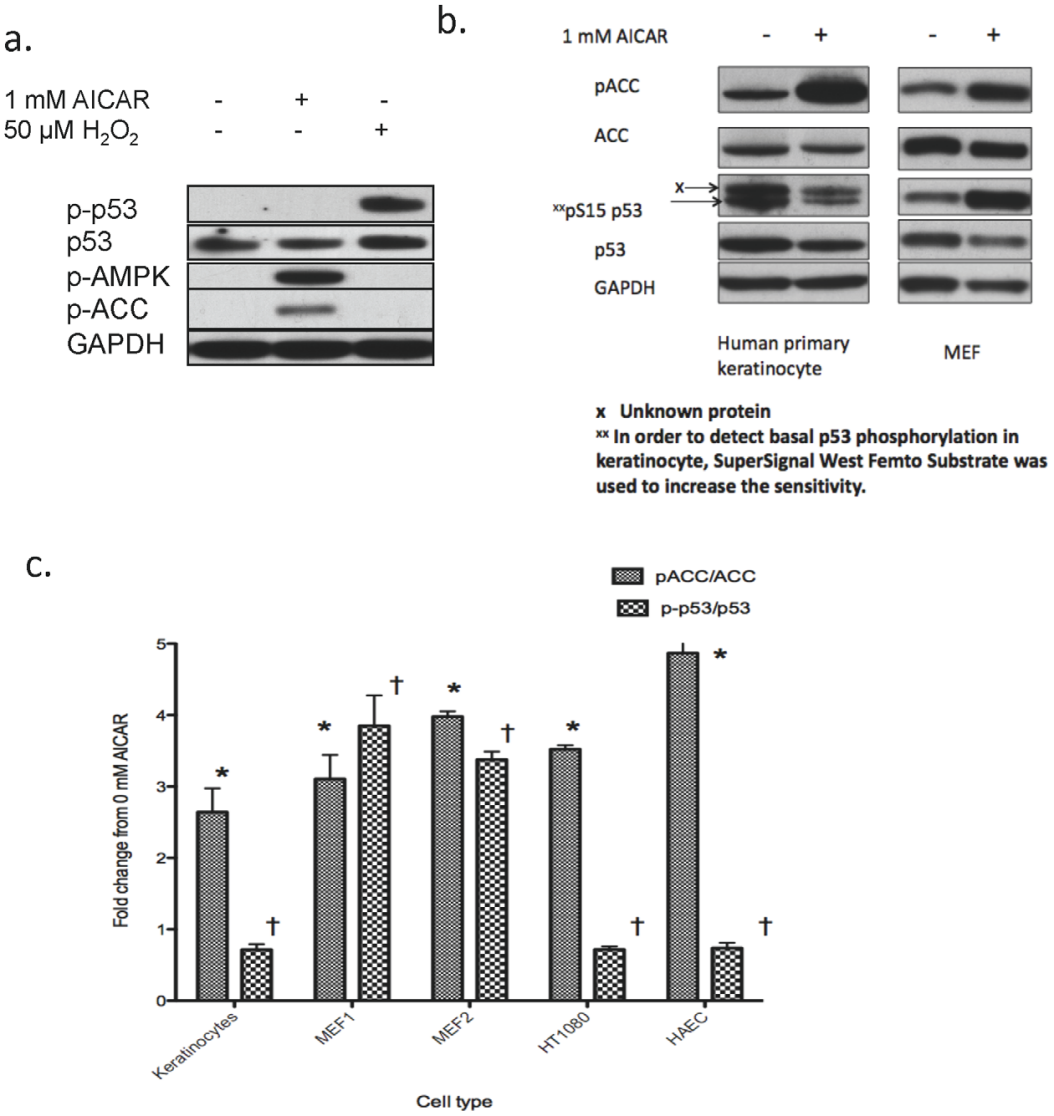
Effects of 1 and 16 hr of AICAR incubation on p53 phosphorylation in keratinocytes, MEFs and other cells. **a.** Primary keratinocytes were incubated with 1 mM AICAR or 50 µM H_2_O_2_ (positive control) for 1 hr. As compared to H_2_O_2_ treatment, incubating the cells with 1 mM AICAR did not increase p53 phosphorylation despite a robust increase in pAMPK and pACC. **b.** Primary keratinocytes and embryonic fibroblasts (MEF), were incubated with 1 mM AICAR for 16 hrs to examine its effects on p53 abundance and phosphorylation at S15. AICAR increased AMPK activity, as evidenced by a 3–5 fold increase in the pACC/ACC ratio in both cells. In contrast, p53-phosphorylation was increased in MEF but was decreased in keratinocytes. **c.** Fold changes in pACC/ACC and p-p53/p53 in human keratinocytes, MEFs, HT1080 human fibrosarcoma cells and adult human aortic endothelial cells (HAEC). Cells were incubated with AICAR for 16 h as in b. Control cells (fold change 1) were incubated with standard medium without additions.

As noted in the Introduction, in cultured embryonic fibroblasts (MEF), activation of AMPK by 1 mM AICAR increases p53 phosphorylation at S15 and accelerates senescence [19]. Since we found here that AMPK activation diminishes H_2_O_2_-induced senescence in keratinocytes, we examined the effects of incubation with AICAR on p53 phosphorylation (S15 for human S18 for MEF) in the two cell types. As shown in Fig. 9A, we did not observe an increase in p53 phosphorylation in keratinocytes after a 2-hour incubation with 1 mM AICAR (Fig. 9a), despite robust AMPK activation as evidenced by specific increases in AMPK and ACC phosphorylation. Consistent with Fig. 7a, 50 µM H_2_O_2_ markedly increased p53 phosphorylation at S15 (Fig. 9a), although it did not activate AMPK (pAMPK). Next, we examined the effects of 16 hours of continuous incubation with 1 mM AICAR, which has been reported to increase p53 phosphorylation in embryonic fibroblasts (MEF) [19]. As shown in Fig. 9b, AICAR increased the phosphorylation of the AMPK substrate ACC in both cells, but it increased p53 phosphorylation at S15 only in the MEF (3-fold, p<0.01, n = 3). In keratinocytes, both the abundance (p53/GAPDH) and phosphorylation (p-p53/p53 ratio) of p53 were decreased by 35% (p<0.01, n = 3). We next investigated this phenomenon in various cell types. As summarized in Fig. 9c, AICAR caused robust increases in p53 phosphorylation (3–4 fold, p<0.01, n = 3 each) in two fibroblast cultures (MEF1 and MEF2), but in other cell types including keratinocytes, human fibrosarcoma cells (HT1080) and adult human aortic endothelial cells (HAEC), it decreased p53 phosphorylation (p-p53/p53 ratio) by 30–35% (p<0.01, n = 3 each). Such diverse results were observed despite similar (3–5 fold) increases in ACC phosphorylation in all of the cell types tested. Collectively, these findings strongly suggest that although p53 can be a substrate of AMPK, the effects of AMPK activation on p53 phosphorylation at S15 are cell-type dependent.

## Discussion

We show here that H_2_O_2_-induced premature senescence in primary human keratinocytes can be prevented by AMPK activation. Thus, we found that: (1) a low dose of H_2_O_2_ (50 µM) activated p53 and secondarily p21^C1P1^ in these cells and subsequently increased SA-Ga1 activity. (2) AMPK activity was not affected by H_2_O_2_ at this low dose, but its prior activation by AICAR or resveratrol prevented these H_2_O_2_-induced changes and (3) AMPK activation appeared to exert these effects, at least in part, by inhibiting p53 transactivation. In addition to these novel actions of AMPK, (4) we demonstrated that AMPK-induced p53 phosphorylation is a cell-type dependent phenomenon.

p53 acting on p21^CIP1^ has been implicated in the pathogenesis of cellular senescence in response to DNA damage, telomere erosion, chromatin perturbation when histone deacetylase activity is diminished, and other stresses [1]. Senescence can also be induced by p16^INK^ induction through p38 MAPK activation, but our data showed this cascade does not appear to play a significant role in the pathogenesis of H_2_O_2_-induced senescence in keratinocytes when stimulated with H_2_O_2_. Our observations are in line with a recent report that demonstrated whole-body elimination of p16^INK^ activated cells has no effect on skin aging in the mouse in vivo, whereas it significantly improves the phenotype of many other organs [3]. In the present study, we found that activation of AMPK by both AICAR and resveratrol failed to diminish the increase in p53 phosphorylation when it prevented the increase in SA-Gal expression caused by low dose H_2_O_2_. Instead, it decreased p53 transactivation (6 hr) and totally prevented the increase in p21^C1P1^. As will be discussed later, this is in contrast to the findings of others in different cell types. Mechanistically, our data suggest that AMPK acutely regulates p53's ability to transactivate its target genes in keratinocytes. How it does so remains to be determined. One possibility is that AMPK, or a factor controlled by it, modifies p53 so that it does not bind to its cis-regulatory elements, such as those found on the p21 promoter (i.e. inhibition of DNA binding). Alternatively, AMPK could phosphorylate and inhibit another molecule that p53 binds to or recruits to promoters and that this prevents p53-mediated transactivation. The latter could include other transcription factors, such as the FOXOs [18], [34] or perhaps members of the RNA Pol II complex required for transcription.

Another possible mechanism of AMPK regulation of senescence could be through oxidative stress. Oxidative damage has been implicated as a major inducer of senescence [1], [35], an observation supported by this study. AMPK activation in turn has been demonstrated to upregulate anti-oxidant mechanisms that diminish H_2_O_2_ (e.g. an increase in catalase [8], [17]), and conversely, repression of AMPK has the opposite effect. Thus we have shown that inhibition or knock-down of AMPK increases oxidative stress in 3T3L1 adipocytes, especially when lipolysis is concurrently stimulated ([36] and unpublished data) and a similar increase in oxidative stress has been observed in endothelial cells in which AMPK is downregulated [13]. Therefore, the increased number of SA-Ga1 positive cells observed after infection with DN-AMPK and AMPK knock-down in the present study could have been due to oxidative stress and conversely, the prevention of senescence by AMPK activation to its anti-oxidant action.

NF-κB activation also has been shown to cause premature senescence in primary keratinocytes [8]. According to Acosta [37] and Kuilman [38] and their coworkers, in oncogene-induced senescence inflammatory processes in which NF-κB and C/EBPβ are affected, contribute to the activation of p53 and induction of p21^CIP1^ by enhancing the expression of chemokines and receptors. This is noteworthy, since AMPK has been linked to suppression of NF-κB signaling. For instance, we have reported that suppression of AMPK by DN-AMPK intensifies the increase in NF-κB signaling induced by TNF-alpha and the fatty acid palmitate in cultured endothelial cells, whereas activation of AMPK inhibits the ability of both molecules to induce NF-κB activation [14].

The results reported here were obtained in primary human keratinocytes in early passage and may not apply to other cells. Thus, several reports have appeared in which AMPK activation was shown to increase rather than decrease cellular senescence. In one of them, Wang et al [27], using human fibroblasts as a replicative senescence model, found that incubation with various AMPK activators, including AICAR for 3–7 days triggered senescence characteristics, such as the acquisition of SA-Gal activity and increased p16^INK4a^ expression, whereas infection with a DN-AMPK decreased SA-Gal activity. In subsequent papers, the authors attributed these events to phosphorylation of importin α by AMPK [39]. They did not evaluate p53 status, thus it is difficult to relate their findings to those of the present study. On the other hand, their data do raise the possibility that AMPK's role may differ in cells (situations) in which senescence is p16^INK4a^-mediated. In another study, Jones et al [19] found that persistent activation of AMPK (days) leads to p53 dependent cellular senescence in MEFs. When examined here, we confirmed this finding (Fig. 9) in two groups of cultured MEFs treated with AICAR. On the other hand, other low passage human primary cells (keratinocytes, HAEC and skin fibrocytes [data not shown]) and NT1080 human fibrosarcoma cells failed to show increases in p53 abundance and phosphorylation when treated with AICAR. Parenthetically, activation of p53 phosphorylation by AMPK has been reported in rat aortic vascular smooth muscle cells, in which it also mediated senescence [40] and in a human hepatocellular carcinoma cell line [41]. Thus, it appears highly likely that the effects of AMPK activation on p53 phosphorylation and senescence are cell- and possibly context-specific.

This dual role of AMPK on senescence that varies with cell type may also apply to some of its other activities. Thus, activation of AMPK has been shown both to inhibit apoptosis in vascular cells incubated with high concentrations of glucose [12], [42] and the fatty-acid palmitate [14] whereas it caused apoptosis in cancer cells treated with such agents as vincristine and doxorubicin [43], [44]. The reasons for these opposite effects of AMPK on apoptosis as well as senescence in different cells remain to be determined.

On the assumption that brief periods of AMPK activation can protect cultured keratinocytes from becoming senescent, a logical question is what is the physiological relevance of this finding. As reviewed elsewhere [1], cellular senescence has become a center of attention in great measure because of its potential links to cancer and aging. With respect to cancer, oncogene-induced senescence has been viewed as a means by which the organism inhibits neoplastic growth and it could be particularly beneficial in young people [1]. As already mentioned, epidermal (keratinocyte) senescence has been noted to increase with age in humans [2]. If so, it would hypothetically protect them against “cancerous” changes and the prevention of senescence by AMPK activators might not be desirable. On the other hand, it has become increasingly clear that senescent cells secrete proteins such as inflammatory cytokines and growth factors that could have tumorogenic and other adverse effects on adjacent cells [1], [37], [38]. If so, the prevention of cellular senescence in skin, blood vessels, pancreatic β-cells and other sites might have important benefits [45], [46]. In skin, the application of topical preparations could have the added potential benefit of not activating AMPK systemically.

In summary, the results demonstrate that premature senescence, induced by low dose hydrogen peroxide in primary human keratinocytes, is closely linked to the activation of p53 and secondarily an increase in nuclear p21^C1P1^. They also suggest that these abnormalities can be prevented by AMPK activators. The physiological relevance of these findings, and their applicability to other cell types, remains to be determined.

## Materials and Methods

### Cell culture

Primary keratinocytes were freshly isolated from infant foreskin and passage 2–4 cells that had not reached terminal differentiation were used, as reported previously [47]. No consent form was needed since keratinocytes were grown from human foreskin tissue that had been discarded at the time of circumcision. Subjects were not identified. The protocol was approved by the institutional review board of Boston University Medical Center. The cells were isolated, selected and cultured in growth media containing serum free MCDB-153 medium (Sigma, St. Louis, MO) supplemented with amino-acids (50 mg/L of L-histidine, 99 mg/L of L-isoleucine, 14 mg/L of L-methionine, 15 mg/L of L-phenylalanine, 10 mg/L of L-tryptophan, and 14 mg/L of L-tyrosine), and growth factors (1 ml/L bovine pituitary extract, 12.5 µg/L EGF, 2.5 mg/L insulin, and 25 µg/L PGE1). The medium was changed every other day. On the day of the experiment, the culture media was changed to basal medium (no growth factors) for 5–6 hrs.

Non-transgenic control embryonic fibroblasts from mus musculus were gifts from Dr. Leonard Guarente at MIT and Dr. David Sinclair at Harvard. They were obtained in separate projects, and cultured in DMEM containing 10% FBS. Human aortic endothelial cells and dermal fibrocytes were purchased from Lonza and human fibrosarcoma HT1080 and NIH3T3 cells from ATCC. All cells were cultured in recommended media.

### Hydrogen peroxide treatment, assessment of cell viability and SA-Gal staining

Cells in basal medium were treated with the indicated concentration of hydrogen peroxide for 2 hrs. After this, they were incubated in fresh growth medium without H_2_O_2_ for the remainder of the study. The precise concentration of hydrogen peroxide in the 30% stock solution (Sigma, St. Louis) was determined spectrophotometrically by measuring its absorbance and extinction coefficient at 220 nm. Cell viability was assessed by incubating the cells at the end of the incubation in a medium containing alamar-Blue (resazurin) dye (Sigma, St. Louis, MO) for 30 min–2 hr and measuring fluorescence at Ex 530 or 560 and Em 590 [48]. The alamar blue method has recently replaced the traditional MTT assay, because of its greater sensitivity and ease of use. SA-Gal staining was done after paraformaldehyde fixation with X-Gal at pH 6 [2]. A total of 300–400 cells in 3–4 wells were evaluated to assess the percentage of SA-Gal positive cells.

### AICAR and resveratrol treatment

AICAR was purchased from Sigma (St. Louis, MO) and a 100 mM solution was freshly prepared in PBS. Resveratrol, obtained from BioMol (Plymouth Meeting, PA), was dissolved in DMSO and stored at a concentration of 100 mM. Indicated concentrations of AICAR or resveratrol were added prior to hydrogen peroxide as described in the text. Two hours after the addition of hydrogen peroxide, the basal culture medium was replaced with fresh growth medium.

### Recombinant adenoviruses and lentivirus

Recombinant adenovirus expressing DN-AMPK was prepared as described previously [14]. Adenovirus with p53 consensus binding sites and a luciferase reporter downstream was created from Pathfinder vector (Clontech). Approximately 20 MOI of the viruses were used to infect one well of cells grown in a 6-well plate. Luciferase activity was assessed as described previously [14]. The recombinant lentivirus plasmid expressing shRNA targeting AMPKα1 (TGAATTAAATCCACAGAAA) and p53 (ACATTCTCCACTTCTTGT) were created as described before [30]. Lentiviruses were packaged in 293T cells and purified using a kit (lentivirus production kit, ATCGbio.com, Vancouver, Canada). Approximately 20 MOI of the viruses with 8 µg/ml polybrene were used to infect one well of cells grown in a 12-well plate.

### Western blotting

Cells were harvested with lysis buffer and subjected to SDS-PAGE and western blotting as described previously [30]. The following primary antibodies were used: pS79 and total Acetyl-CoA carboxylase, pT172 AMPK and AMPK, pS431 LKB1, p38MAPK and p21^CIP^ (Cell Signaling Technologies). LKB1 and p16 ^INK^antibody was obtained from Santa Cruz Biotechnology.

### Statistics

Statistical analysis was performed by one way ANOVA and a post-hoc Dunnet test using GraphPad Prism (GraphPad Software, La Jolla, CA). P less than 0.05 was used as an indicator of statistical significance.

## Supporting Information

Figure S1 Keratniocytes were infected with an adenoviral vector expressing GFP at 40–50% confluency. No morphological changes were observed as a result.
(TIF)
