# Supplementary figures and images for: Acute Activation of AMP-Activated Protein Kinase Prevents H2O2-Induced Premature Senescence in Primary Human Keratinocytes
Source: PLoS One. 2012 Apr 13;7(4):e35092. doi: 10.1371/journal.pone.0035092 (PMC3325987; doi:10.1371/journal.pone.0035092)

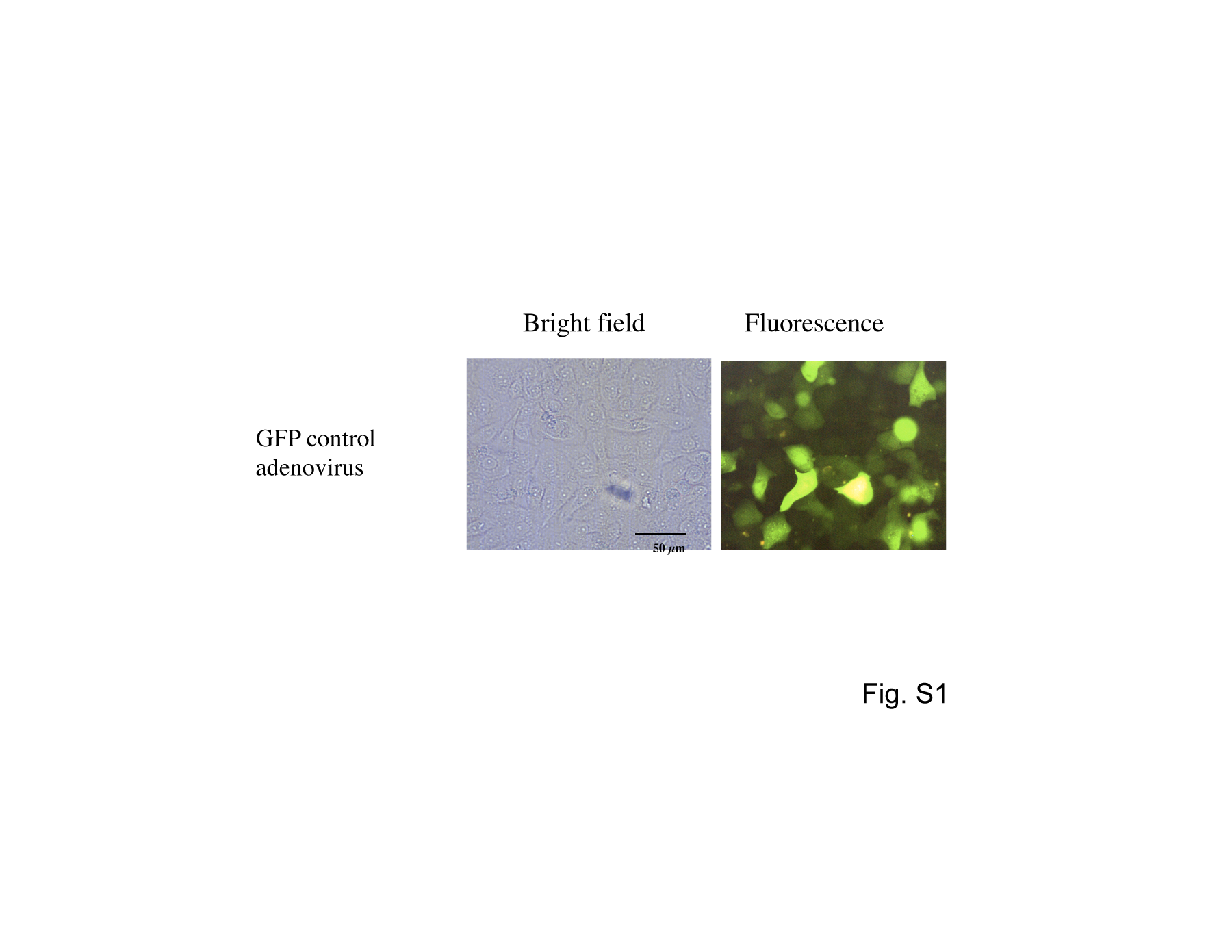

Supplement: Figure S1 — Keratniocytes were infected with an adenoviral vector expressing GFP at 40–50% confluency. No morphological changes were observed as a result. (TIF) [file pone.0035092.s001.tif]
